# ‘Beyond the Reach of Palliative Care’: A Qualitative Study of Patient and Public Experiences and Anticipation of Death and Dying

**DOI:** 10.1177/10497323241246705

**Published:** 2024-06-21

**Authors:** Kristian Pollock, Glenys Caswell, Nicola Turner, Eleanor Wilson

**Affiliations:** 1 School of Health Sciences, 6123 University of Nottingham, Nottingham, UK

**Keywords:** death and dying;, palliative care;, qualitative research;, the good death

## Abstract

The demands and costs of health care resulting from increasingly ageing populations have become a major public health issue in the United Kingdom and other industrially developed nations. Concern with cost containment and shortage of resources has prompted a progressive shift in responsibility from state provision of care to individual patients and their families, and from the institutional setting of the hospital to the domestic home. Under the guise of choice and patient centredness, end-of-life care is framed within a discourse of the ‘good death’: free from distress and discomfort and accompanied by significant others in the preferred place, usually assumed to be home. The promotion of the ‘good death’ as a technical accomplishment enabled by pre-emptive discussion and advance care planning has sidelined recognition of the nature and significance of the pain and suffering involved in the experience of dying. There has been little research into the disparity between policy and professional assumptions and the lived reality of end of life. In this paper, we present findings from a qualitative study of how terminally ill patients, bereaved family members, and members of the public understand, anticipate, and experience death and dying. These findings contribute to an important and timely critique of the normative idealisation of death and dying in health policy and practice, and the need to attend closely to the real-world experiences of patients and the public as a prerequisite for identifying and remedying widespread shortcomings in end-of-life care.

## Background

Health policy and professional practice aim to minister to the existential as well as corporeal challenges of dying in modern industrial societies in which the resource demands and escalating costs of end-of-life care have become a major public health issue (Bone et al., 2018; Giorghiou & Bardsley, 2014; Watt et al., 2023). However, concern with cost containment in the delivery of care has sidelined recognition of the nature and significance of the pain and suffering which can be involved in the experience of death and dying (Baddeley et al., 2022; Bolton et al., 2022; Rattner, 2019; Ruijs et al., 2013; Salès-Wuillemin et al., 2023). End-of-life care is framed within the discourse of the ‘good death’, promising comfort, control, and personal choice as an outcome of pre-emptive discussion and advance care planning (ACP) (Borgstrom, 2015; Collier & Chapman, 2023; Cottrell & Duggleby, 2016; Hansford et al., 2022) and through open awareness and acceptance of death (Livne, 2019; Zimmerman, 2012). Social marketing strategies such as the Dying Matters Coalition in the United Kingdom (https://www.hospiceuk.org/our-campaigns/dying-matters) and Dying to Talk in Australia (https://www.health.gov.au/contacts/dying-to-talk) aim to encourage the public to overcome their assumed resistance to talking and thinking about death and dying in order to engage in active discussion and planning for their future demise. ACP is widely promoted as a way to encourage individuals to discuss, anticipate, and, ideally, document their treatment preferences and goals of care as they approach the end of life (Rietjens et al., 2017; Sudore et al., 2017). Through the exercise of choice, individuals are promised ‘a good death’, free from distress and discomfort, in their preferred place and accompanied by the persons of greatest significance to them: nothing to be afraid of (Leiter, 2021; Mannix, 2017). Home deaths are assumed to be the preferred option of most patients and to have the considerable advantage of avoiding the cost and distress of prolonged and often futile active treatments such as cardiopulmonary resuscitation, ventilation, or artificial hydration and nutrition. These are widely perceived to be hazards of dying in hospital (Kaufman, 2006; Luta et al., 2020; Robinson et al., 2016).

The relocation of end-of-life care from hospital to community care settings involves a huge shift in responsibility and costs from state to patients and their informal carers (Gardiner et al., 2020; Marie Curie, 2019; Rowley et al., 2021). Even if death eventually occurs in an institution, most of the time that people are dying is spent where they usually live. The greater part of care and support is provided informally by family, friends, or neighbours (Broese van Greonou & De Boer, 2016; Funk et al., 2010; Martz & Morse, 2017). The provision and quality of professional end-of-life care is variable in community and institutional settings, particularly in relation to patients who experience social and economic disadvantage or minority group status (Deri Armstrong & Devlan, 2022; Dixon et al., 2015; Hansford et al., 2022; Rowley et al., 2021). The discourse of ‘the good death’ reflects the holistic values of hospice and palliative care developed from the mid-1960s and remains oriented largely to patients dying from cancer (Clark & Seymour, 1999; Doyle, 2005; Tobin et al., 2022). However, many deaths among older people are from diseases other than cancer and occur following a prolonged period of decline characterised by comorbidity and increasing frailty (Teggi, 2020; Watt et al., 2023). These deaths tend to be more difficult to anticipate or ‘plan’ for than cancer (Orlovik et al., 2023; White et al., 2016). Despite the reassuring tropes generated by the discourse of ‘the good death,’ the reality for many dying patients and their families is variable, if not poor, care accompanied by the experience of pain and suffering (Baddeley et al., 2022; Benson et al., 2018; Bolton et al., 2022; Ruijs et al., 2013). Whilst many assumptions have been made about patient and public preferences and that these generally correspond to notions of ‘the good death’, there is a notable lack of research into what people *think* about death and dying, their *experience* of end-of-life care for friends and family members, or their *hopes and anticipation* for the future (Cox et al., 2013; Islam et al., 2021; Robinson et al., 2016). The nature and reality of suffering has been largely sidelined (Baddeley et al., 2022; Bolton et al., 2022). Better understanding of these experiences is a prerequisite for developing genuinely responsive, patient-centred care (Baylis et al., 2023; Black et al., 2018; Hansford et al., 2022).

This paper presents findings from a qualitative study which aimed to explore patient, family, and public perspectives of death and dying, and how these correspond to policy and professional stereotypes about ‘the good death’ and the importance of planning for the end of life. It focuses on participants’ accounts of their experience of witnessing the death and dying of those close to them, and how this shaped their expectations and anticipation of their own. These findings contribute to an important and timely critique of the normative idealisation of death and dying in health policy and practice, and the need to attend closely to the real-world experiences of patients and the public as a prerequisite for identifying and remedying widespread shortcomings in end-of-life care (Collier & Chapman, 2023).

### Theoretical Framework

The underpinning theoretical basis for the study is social constructionism (Berger & Luckmann, 1966). This views knowledge and understanding of the world as co-constructed through the shared meanings which people negotiate in social encounters and through which they construct and maintain a sense of shared reality. This is at the same time experienced as ‘taken for granted’ but also dynamic and shifting. Within this paradigm, participant responses do not involve ‘description’ of pre-existing and stable thoughts and opinions. Rather, the accounts produced in different settings, including clinical consultations and research interviews, are responses to specific social situations and influenced by context and purpose of the encounter.

## Method

The study was based on a series of deliberative discussion groups (DDGs) (Rothwell et al., 2016) with members of the general public and qualitative interviews with bereaved family members (BFMs) and patients who were aware of their terminal illness and limited prognosis. Each DDG met on four occasions, at roughly weekly intervals to consider a specific topic: ‘Talking about death and dying’, ‘The good death’, ‘Choice and future planning’, and ‘Compassionate Communities’. The first three of these topics were selected as key components of the current discourse of ‘the good death’ which we wished to explore in the study. Compassionate Communities was chosen as an approach to exploring alternative models of end-of-life care which have been proposed to improve the experience of death and dying in future. Participants were sent several resources (including links to short videos, media articles, and discussion pieces) to review prior to each meeting. These were intended to provide some background information about each topic and inform discussion. The aim of the DDGs was to provide an opportunity for participants to develop their understanding of the topic through a series of iterative discussions in which they reflected on, and learned from, their shared experiences with fellow participants. Interviews with BFMs explored their experiences of witnessing and/or providing end-of-life care for a deceased friend or family member. Interviews with terminally ill patients explored their perspectives of death and dying and anticipation of their future. A topic guide provided an aide memoire and means of loosely structuring a discussion which was led by participants’ experiences and the issues they considered to be of greatest significance and concern.

### Recruitment

The deliberative discussion group and most (26) BFM participants were recruited through information about the study placed in a range of media, including Twitter, Facebook, and Call for Participants, as well as in local and national organisation newsletters and magazines. A further eight BFM participants and all 12 patients were recruited through two local hospices where staff identified eligible participants and provided information about the study. Following receipt of information, participants who were interested in taking part contacted the researchers.

We received expressions of interest from considerably more BFMs and participants wishing to take part in a DDG or individual interview than we were able to include in the study. Consequently, we adopted an increasingly purposive approach to recruitment as the study progressed to increase diversity, particularly in relation to age (targeting younger participants), ethnicity, and sex (targeting men). We limited the number of participants who were members of organisations such as Compassion in Dying and/or held strong views about assisted dying and euthanasia so that these perspectives were included but without dominating the study.

### Data Collection

Interviews were conducted by four female researchers with academic backgrounds in sociology and medical anthropology. Written or recorded consent was obtained from all participants prior to the start of the interviews. In response to the lockdown following the COVID-19 pandemic, data collection moved from being face to face to being undertaken by phone or online. All but two DDG sessions were held by video link. Five participants who wished to take part in the discussion but were not able, or did not wish, to join a group took part in a single interview covering the same topics as the DDGs. Ten BFM interviews were face to face. One patient and one BFM participant opted to complete the interview through a series of emails. All other patient and BFM interviews were held over the phone (18) or via video link (16). All interview participants are referred to by pseudonyms. Patient participants are designated by title and surname (e.g. Mr Jones) and bereaved family members by first and surnames (e.g. Carrie Nolan). DDG participant data extracts are designated by the group number, the number of the group session in parenthesis, whether the speaker was male or female, and the number assigned to each participant within each group (e.g. DDG2(1)F5).

### Analysis

Interviews and DDG meetings were recorded and transcribed by a professional transcription service, checked, and anonymised. The qualitative software analysis programme NVivo12© was used to facilitate analysis and management of the data (Bazeley & Jackson, 2013). A thematic analysis of the transcripts was undertaken through an iterative process including constant comparison (Braun & Clarke, 2019; Charmaz, 2006). Each transcript was independently coded by at least two members of the research team, and the development of the coding frame and identification of themes were discussed at regular team meetings. Separate coding frames were developed for each of the three data sets relating to DDGs, patients, and BFMs. These reflected the different issues and concerns discussed with each group of participants. The material relevant to specific topics was scattered throughout the discussion and gathered together throughout the process of coding and analysis. The same content could be assigned to more than one code. Overarching themes were developed from the identification and organisation of related codes through a flexible and iterative process with an interpretive focus on what was most significant and meaningful to our participants.

### Ethical Considerations

Research in palliative and end-of-life care raises ethical concerns about the distress which might be caused in discussing painful and challenging topics and experiences, and the appropriateness of asking people who are gravely ill to take part in interviews. However, we believe that research participation should be as inclusive as possible and the agency of participants in making decisions about research participation should be respected (Emanuel et al., 2004; Gysels et al., 2008). The researchers were experienced in conducting interviews about sensitive issues and alert to signs of distress. The information provided to potential participants asked them to consider carefully how they would feel about discussing issues relating to death and dying of themselves and others before deciding to take part. Participants were assured that they could pause or end the interview at any time, although none did so.

The study received approval from the University of Nottingham Faculty of Medicine and Health Sciences Research Ethics Committee (BFMs recruited as healthy participants) and from a Health Research Authority Research Ethics Committee (patients and BFMs recruited through the NHS and independent hospices).

## Results

Data were collected between October 2019 and February 2021. The 34 BFM participants ranged in age from 19 to 82, although only four were aged under 50 and two over 80. The majority (24) were women. The primary cause of death of half (23) of the BFMs’ deceased relatives was cancer. A wide range of causes of death was distributed among the remaining 24 (Table 1). Twelve patient participants were recruited, ranging in age from 58 to 88. Seven were women and eight had a primary diagnosis of cancer (Table 2). Forty-one participants took part in one of seven DDGs or five individual interviews. DDG participants ranged in age from 36 to 81 years and most (32) were female (Table 3). Coded material from each data set relevant to the topic ‘experience and anticipation of death and dying’ was identified as a subset of relevant data for analysis in this paper and organised under four broad themes presented below: ‘Accounts of death and dying’, ’Anticipation of death and dying’, ‘The unpleasantness of death and dying and of being a witness to suffering’, and ‘Beyond the reach of palliative care’.

**Table 1.** Characteristics of Bereaved Family Members.

Characteristic		Deceased Relationship to participant	
Identified gender		Father	5
Female	24	Mother	12
Male	10	Husband	6
Total	34	Wife	7
Age		Grandmother	3
19–29	2	Father-in-law	1
30–39	0	Daughter-in-law	1
40–49	2	Sister	1
50–59	6	Uncle	1
60–69	14	Friend	8
70–79	6	Friend’s child	1
80–89	2	Stranger	1
Not known	2	Total*	47
Total	34		

Primary identified cause of death of deceased person		Place of death	
Cancer	23	Home	14
Dementia	2	Hospital	11
Renal failure	2	Care/nursing home	8
Pneumonia	4	Hospice	8
Cardiac arrest	1	Outdoors	1
Heart disease/failure	2	Dignitas	3
Stroke	3	Not known	2
Multiple sclerosis (MS)	2	*Total	47
Progressive supranuclear palsy (PSP)	1		
Frailty	1		
Sepsis	1		
Anorexia	1		
Accident	1		
Multiple co-morbidities	1		
Unknown	2		
*Total	47		

^*^The total exceeds the number of participants because some BFMs talked about more than one death

**Table 2.** Characteristics of Patient Participants (*N* = 12).

Gender	
Male	5
Female	7
Age	
50–59	1
60–69	1
70–79	5
80–89	5
Primary diagnosis	
Cancer	8
Neurodegenerative disease	2
Heart disease	1
COPD	1
Preferred place of death (if possible)	
Home	7
Hospice	3
Hospital	1
Don’t know/no preference	1

**Table 3.** Deliberative Discussion Group and Interview Participants.

Total no. of participants (*n* = 41)
Deliberative discussion group participants (*n* = 36)
Individual interviews (*n* = 5)
Age range: 36–81 yrs
30–49 yrs, *n* = 15 (34%)
50–69 yrs, *n* = 16 (39%)
70–89 yrs, *n* = 10 (24%)
Identified gender
Female (*n* = 32)
Male (*n* = 9)
Marital status
Single (*n* = 4)
Married/partner (*n* = 22)
Divorced/separated (*n* = 12)
Widowed (*n* = 2)
Not known (*n* = 1)
Education
Secondary (*n* = 7)
Tertiary (*n* = 29)
Not known (*n* = 5)

### Accounts of Death and Dying

Participants’ accounts included a wide range of different experiences of death and dying as well as observations regarding ‘types’ of death and imagining their own. Some positive experiences corresponded to the ideal of the ‘good death’: dignified, comfortable, pain free, in a preferred place, and accompanied by the persons of greatest significance to the deceased.

My mum, my auntie, my uncle, my granddad, me and the carer … were in the room, and she just stopped breathing really calmly, really peacefully. There was no horrendous clutching at the chest or pain or anything, it was very peaceful and that was it, she just slipped away really peacefully. (Carrie Nolan – death at home)

So, yeah, it was a beautiful kind of wonderful poetic death, because all of her children were there. … So, we all got there before the last breath. The nurses came in, I was talking to her. I was just stroking her head and just saying, “It’s OK mum, you can go. It’s all right, we’re all here, we’re all going to look after each other” …. I was just talking her through those last moments, really, so that she wasn’t dying in silence … not knowing what was happening. (Heather Smith – death in hospital)

Untimely and traumatic deaths were contrasted with those that occurred predictably, and relatively quickly and calmly, at the end of a long life.

And I think some deaths are always, people that die in horrific accidents and things, it’s always going to be awful. But, actually, it doesn’t, when it’s an illness or something or like old age, it doesn’t have to be horrid. It can actually be quite dignified and very peaceful. (Carrie Nolan)

My mum was 83, she’d had a good life, her body just failed her in the end, is very different to when somebody dies in either a traumatic way or a sudden way, and you’re not actually prepared for it. (Helen Bryant)

Some people looked to the experience of family members as a good augur of what might eventually happen to themselves. When anticipating their own death, participants tended to exhibit a degree of optimism, although some considered that, regardless of preference, the nature and manner of dying was a matter of fate.

And the actual act of dying. I’ve never wanted a messy death as I’ve always called it. All my grandparents just suddenly dropped down dead from a heart attack, and I just always assumed that was the way I was going to go. (Mrs Tomkins)

Well, it’s like if you could put the lights out and that would be it; that would be my ideal way of going. You go to bed and just go to sleep and not know any more. That would be the best way. Not a painful death or an accidental death, one where you’re injured. You know, it’s just fate, what happens is fate, and that’s what we get. (Mr Gordon)

As the preceding extracts illustrate, participants often wished for a quick, unexpected, and ‘clean’ death, unequivocally avoiding the degradation and indignity of the ‘messy’ experience they had witnessed in others.

If I had to die, I would choose for it to be through some kind of accident rather than just seeing myself decline over time. I think that’s the most, I don’t want to grow old if you know what I mean. I don’t want to get to that stage …. No, it would just be completely random. I wouldn’t want to know when it was going to happen, it would just happen. (Lynn Fraser)

If I just drop dead with no warning whatsoever of a massive heart attack or a massive stroke and that is it, dead, that’d be fantastic, because I wouldn’t know it was coming … If you could be fit and healthy, going about your daily things that you do and then you just drop dead, fantastic. That would be the best possible. (Felicity Burgess)

However, as Felicity Burgess recognised, personal preference for a quick and unexpected end was often tempered by awareness of the trauma such deaths occasioned for family members and others left behind, who had no chance to anticipate or prepare for the event.

It would just come out of the blue and that would be that. It’d be horrible for the family, but for me it’d be fantastic.(Felicity Burgess)

DDG6(1)M1: It’s really interesting, isn’t it, because of course you talked about if you could choose the way you’d go it’d be over like that. I’d like to use a sniper who’s a really good aim and then I could just be gone completely. So, for me, that’s probably a good way to go, but for my family they would have had no time to prepare, it would have been a complete shock.

Concern for others was frequently expressed and points to the strongly relational nature of death and dying. Dying persons and family members endeavoured to subordinate their own interests to promote those of others, alongside an awareness of the very considerable challenges that could be involved for all concerned.

DDG7(4)M2: I think the person who is going through [dying], they will have two things in their mind from my experiences, … like one is, do they want to really go through the pain, what is the treatment going to be? The other thing that goes in their mind, which they usually don’t tell anyone, is how it is going to impact others, are they going to be a pain for others, for the loved ones, and that always goes through their mind …. They always think about the loved ones, that’s the first thing, more than the pain, that’s what I observed with my wife.

DDG6(1)M1: I think for me I worry about having a prolonged, drawn out, agonising end … and so it’s that that I worry about. It’s the pain and that, and you don’t want your children to see you be ill for a long time.

The nature of a ‘good’ death varied according to the circumstance and perspective. People did not necessarily have fixed or certain ideas about this. Sudden death was recognised to be good from the individual’s perspective but traumatic for those around them. Precise prognosis might be unwelcome for the dying person but welcomed by family members who needed to manage resources and expectations and plan for the future.

DDG6(1)M1: If you’ve got somebody who’s terminally ill and you know that their prognosis has been six months, for example, it might not be great for the individual because they’ve got that ticking clock knowing that they’re going to get closer and closer to the last day, but for everybody else around we can feel in a bit more control, we can start to prepare, we can start to get arrangements in place. So, I don’t know which way is best or worst, but I think there’s a really interesting difference, isn’t there, depending on the perspective that you have of it.

Death occurring in great old age was generally considered less troubling and, to an extent, to be expected. Some participants tried to find a positive gloss on the death of their loved ones, but regardless of place or accompaniment, the harsh reality of the suffering often experienced during dying was inescapable.

### Anticipation of Death and Dying

Patient participants tended to be pragmatic in their anticipation of the future and a life expectancy they knew to be limited. Discussion of this topic with others appeared to be uncommon, including with health professionals. Information was not necessarily sought or wanted, although several patients and BFMs described frustration and disappointment that they had not been able to find out more about the nature and timing of their own or their relatives’ dying.

I mean the doctor did tell me that I’d do more sleeping, the sleeping would increase. He did tell me that, he said you’re going to sleep more. I said that’s all right, I don’t mind sleeping. (Mr Jones)

Well, I’ve asked, and it’s a bit like saying, “Can you give me a prognosis?,” and no one’s willing to do that. They come back and say, “Well, how long is a piece of string?” .... But I think most people know that if you’ve got cancer there will be pain coming, and I would like to be told that we can manage that, and you won’t have to suffer much or for long. Yeah, I think I would like someone to tell me that. (Mrs Prentice)

Acknowledgment or discussion of impending death was not always felt to be necessary or welcome. Some patient participants did not express a great desire for knowledge about their prognosis and what the experience of dying was likely to involve, preferring to take one day at a time and leave the future to take care of itself. Indeterminacy and uncertainty could be preferred to too much precision.

Nobody’s said anything else, and so I assume from that, I ought to ask I suppose, but do I really want to know, how long, do they know? I don’t know. So, again do I really want to know? This is something I keep throwing around in my mind, do I want to know how long, is it important, I don’t know. I’m still throwing it around. (Mrs Wood)

For many participants, it was anticipation of the experience of dying that was troublesome, and not being able to know what this would be like, rather than death itself.

DDG2(1)F4: Yeah, that’s made me think that there is a fear there. I think that you don’t know if your decline is going to be agonisingly slow or whether or not it’s going to be, you know, just like that and one day you’ll go to bed and you won’t wake up the next day.

She cried two or three times. Sometimes she’d sit there, and she’d start crying. Just tears rolling down, she never wailed or anything. I’d say, “What’s wrong?”, She’d say,“ I’m scared.” I’d say, “What are you scared of?”. “ I’m scared of what’s going to happen, what’ll happen to me? What will it be like when I die?” (Oscar Reed, death at home)

However, the process of dying was widely anticipated to be unpleasant and a subject arousing apprehension and fear.

### The Unpleasantness of Dying and of Being a Witness to Suffering

Participants described diverse experiences of death and dying, ranging along a spectrum from ‘good’ to ‘bad’ and in a range of settings. However, fear and apprehension were common, based partly on lack of knowledge and uncertainty, but often more concretely, on the experience of witnessing the suffering of others. Even those who recounted a positive experience, in which the dying person was peaceful and comfortable in the hours or days prior to death, also described the awfulness of the period of pain and decline which had often preceded this, and the unexpectedly visceral and shocking nature of the dying person’s physical deterioration. Many accounts focus on the horror of an experience of witnessing dying, especially when this had been protracted, involved pain, suffering, and loss of control and cognition.

One night I remember she [mother] phoned me up very distressed. She says, “Oh, I don’t know what’s happening, there’s water coming out my legs.” I said, “What do you mean, there’s water coming out of your legs?” She says, “I’m having to put towels around my legs.” And I didn’t know things like that happened, it was horrible, it was horrible, and she was in a terrible state. ... So yeah, as I say it was just a horrible, horrible year and I think a horrible end for my mum. (Felicity Burgess, death in care home)

So, I went behind the curtain and just, she was lying with her mouth open. I thought I was going to remember that noise for the rest of my life, and I can’t remember it anymore, which is really good. Because you take that death rattle away with you, and it echoes in your head for weeks. It just echoes in your head, it’s all you can hear. And the smell of the body, it’s all you can smell. You can taste it in the back of your throat. And it’s traumatic. It’s absolutely traumatising. And it’s an embodied trauma that you can’t let go of. You can’t forget and you live it every single day. ... But I still, I can just be doing the washing up and I will relive those images and those sounds and the smell. ... And I think when they, oh god it was awful, when they moved her body all the fluids came out all over her clothes. (Heather Smith, death in hospice)

I don’t know what it was, I don’t know whether it was the tumour, but there was, the stench was absolutely awful …- of rotten flesh. … Because the cancer, I mean it was incredibly painful for her, because the tumour was massive, absolutely massive …. So, it was open, weeping, bleeding, and it was huge. So, the pain was absolutely intense. (Amy Watkins, death at home)

Some participants described the opportunity to accompany their loved one to the end of their life as a positive and privileged experience. Others found the event, and the witnessing of their relative’s deterioration and suffering, to be extremely distressing. In some accounts, a sense of privilege in accompanying their loved one to the end of life coexisted with the memories of trauma and bewilderment at the deeply unpleasant sights, sounds, and smells of dying.

### Pain

The occurrence of pain among dying relatives and the anticipation of pain during the experience of their own dying was a widespread concern among participants.

So, I don’t want a thought of it being very painful, and that scares me. I know that you can get nurses to come in, and they give you this, there’s a machine at the back end of everything that releases morphine I dare say on a regular basis, and I’m hoping that will happen and I won’t feel too much pain. It’s the pain that I’m scared of more than anything. (Mrs Tomkins)

Pain relief, and particularly morphine, was a source of considerable ambivalence – valued for its ability to bring comfort, but at a cost of consciousness and agency. Several dying relatives were described as resisting the offer of pain relief as they wished to remain conscious for as long as possible. Others were reported to have lost consciousness some time before death and been basically ‘out of it’ during their last days or hours of life. Some participants expressed distress that their relative had been in a state of sedation prior to death and unable to communicate or be aware of their presence.

Which is why she was so out of it, because she was on quite a high dose of morphine just to keep her comfortable. But it took a higher and higher dose to keep her comfortable and obviously she was a bit out of it towards the end. (Carrie Nolan, death at home)

And she didn’t want to be dosed up with morphine because I suppose the thing is, well the more you have that then the more it takes away your capacity to be engaged with the world and, you know, what have you. So, she was definitely anti going down the morphine route. (Amy Watkins, death at home)

DDG2(2)F4: I don’t have any issue with increasing pain relief to make sure somebody’s free of pain if that shortens life, because what’s the point of prolonging it if somebody’s in pain?

DDG2(2)F3: Yeah.

DDG2(2)F4: But I just don’t think that that always happens for various reasons and for me that would be a very frightening thing to be in pain at the end of my life.

DDG2(2)F3: Yes, horrible.

DDG2(2)F3: That definitely is, I think, most people’s worst scenario.

DDG2(2)F4: Absolutely, yeah.

DDG(2)F2: And for the relations too.

The experience of prolonged pain and suffering was extremely distressing and felt to be diminishing, eroding the dignity of the dying person. This could give rise to intense and conflicting feelings of family caregivers wishing for the hastened death of a deeply loved friend or relative.

DDG6(1)F1: I think seeing, so thinking of, my dad passed away recently. I was desperate for my dad to die. That sounds awful, but he hadn’t had a life for so long it was almost cruel. If he’d been a dog, we’d have put him down years ago and we can’t do that, so you make the best of it. So, my dad now being at peace I’m like, oh yeah, that’s so much better.

I was so angry that someone you love so much could be in such agony and you couldn’t do anything about it. All she kept saying was, “Just let me overdose.” And of course, the stuff, the morphine is limited to how much, for obvious reasons, which made it even worse. If only they could have let the limit be unlimited, she would have gone weeks before. But there we are. (Rob Haywood, death in hospice)

A few participants expressed a wish, and in some cases, an intention, to resort to assisted dying: ‘going to Dignitas’, as a means of obtaining release from pain and suffering that was, or might become, unbearable. Several participants talked about people they had known who had done this. Several also felt that a consequence of increased pain relief legitimately administered by health professionals to relieve symptoms for their dying relative had, as a consequence, precipitated death. Others, as in the case of Rob Haywood, wished that this had been an option.

### Beyond the Reach of Palliative Care

Within the diversity of participants’ accounts were many describing deeply distressing and difficult experiences of death and dying and consequent apprehension about the prospects of their own. In some cases, palliative and end-of-life care was found to be wanting through a failure in access and coordination of effective care.

And I think it’s all very well because everybody says, “Oh palliative care is wonderful!” It is not! I mean even fighting [sister’s] corner it was crap. You know. And we say …, a chain’s only as strong as its weakest link. And, unfortunately, we’ve got an awful lot of weak links, you know. And no, so, terrified, terrified at the thought of it [death and dying]. Absolutely. (Amy Watkins, death at home)

But when she came back home out of the hospice and started going downhill, there was, it was like this thing about she doesn’t have to suffer any pain. No, that never happened. I look back now and I’m thinking to myself what about that night when this happened, and what about when we had to wait two hours for that, what about when they had to, the nurses came out and they couldn’t do that, and what about when you couldn’t get hold of them drugs? (Oscar Reed, death at home)

Only one participant explicitly related the quality and availability of good care to socioeconomic advantage.

And whatever they say, like Marie Curie and people about the End-of-life, they say, “Oh, but you don’t need to be in pain at the end of your life, we can control the pain.” I think if you get really good treatment and you’re very middle class and you can afford lots of care or whatever or in the right place, the right hospice that can do that. But if you’re not .... (Felicity Burgess)

Other participants concluded that the pain and suffering endured by their relative simply transcended the capacity of even the best professional care.

And he was, then went to the Hospice, pain management, and they couldn’t do anything apart from pump up his morphine. … When you decide that you can’t take this pain, he was beyond the remit of the hospice, OK? Palliative care, he was one of those that palliative care, he was beyond the reach of palliative care. ... Because palliative, the Association of Palliative Care and the associations of the hospices are adamant that they can deal with everything but they can’t and they know they can’t. There are some people who are beyond it. (Mark Hill, death at Dignitas)

In some cases, it was recognised that even the best care and most powerful pain relief could not overcome the intractable force of terminal disease.

## Discussion

Participants described diverse experiences of death and dying across a range of settings, mainly home, hospital, and hospice. Some deaths were described as peaceful and comfortable, especially among older people who had reached the end of a long life. However, participants expressed concern about the process of dying and the uncertainty of what would happen, particularly a fear of prolonged suffering and distress, and lacking capacity and function. Fear most commonly centred on the process of dying, not on death itself. Consequently, sudden death, or failing that, assisted death, was often preferred (Hughes et al., 2008). Witnessing the death of a deeply loved family member could be at the same time a ‘privileged’ experience but also traumatising (Benson et al., 2018). Participants described the deeply distressing embodied memories that lingered after difficult and distressing deaths. Such experiences inhibit the reconstructive effort by which bereaved relatives engage in emotional work of making death good and endeavour to protect the memory and legacy of the deceased by constructing narratives of at least ‘a good enough’ death (Engleke, 2019; Sinding, 2003). Graphic accounts of the prolonged and miserable experience of death and dying, for both patients and the family members who were charged with providing care, highlight the distress intrinsic to such experiences as well as the limitations of palliative and end-of-life care in relation to enablement of ‘the good death’. These were reported to result from fragmented, patchy, poorly coordinated, and inaccessible services as well as the intrinsic challenges of effective control of severe and intransigent symptoms (Baddeley et al., 2022; Barker et al., 2021; Funk et al., 2023; Michaels et al., 2022).

The relational nature of dying was a strong theme throughout all accounts, alongside mutual concern about the negative consequences of terminal illness on others, especially if this involved a prolonged and substantial burden of care and suffering (Broom & Kirby, 2013; McPherson et al., 2007; Rodriguez-Prat et al., 2017). The findings echo those of other studies reporting participant concerns about the transgression of boundaries which may result from intensive care of dying relatives and the desire to protect the legacies and relationships which would be jeopardised by witnessing the patient’s decline and suffering (Benson et al., 2018; Broom & Kirby, 2013). Auriemma et al. (2022) reported that the most commonly expressed attributes of patients’ perceptions of states worse than death were perceived burden on family members and friends and relational disconnection. It is evident that many people prioritise relational decision-making rather than personal choice and autonomy in making plans for future care (Broom & Kirby, 2013; Funk et al., 2023; McPherson et al., 2007; Rodriguez-Prat et al., 2017). This contrasts with the focus of the ‘good death’ discourse on the experience of dying as the outcome of personal choice and individual autonomy through forward planning. This discourse not only ignores the consequences for informal caregivers but also the wider structural determinants of opportunity such as socio-economic status, material circumstances and social resources, diagnosis, or the variable quality and availability of health and social services (Collier & Chapman, 2023; Richards, 2022; Rowley et al., 2021). In the real world, family members confront not only the burden of care but also the bureaucracy resulting from the provision of care within a complex but poorly resourced and uncoordinated system of health and social care services (Barker et al., 2021; Briggs, 2010). Rather than being the consequence of ‘choice’ or (lack of) future planning, drastic differences in the experience of dying result from the perpetuation of deeply entrenched inequality and differences in social, material, and economic resources available to patients and families (Deri Armstrong & Devlan, 2022; Quinn et al., 2023; Richards, 2022).

Like all discourses, the idyll of the ‘good death’ privileges some issues (choice, control) and sidelines others: notably, reference to the enormous challenges and adverse consequences for family caregivers and the existential suffering involved in death and dying (Baddeley et al., 2022; Bolton et al., 2022; Broom et al., 2016; Cottrell & Duggleby, 2016). In particular, the discourse ignores the sheer physicality of dying, the sights and smells, and embodied memories resulting from the reality of prolonged physical pain and bodily disintegration of the unbounded body (Harrop et al., 2016; Lawton, 2000; Ruijs et al., 2012). Moreover, the moral obligation of informal caregivers to commit to supporting patients dying at home writes out the effort and ambivalence which may often be felt but cannot easily be expressed (Benson et al., 2018; Broom et al., 2016; Harrop et al., 2016; Mehta et al., 2014). In documenting participants’ accounts of pain and distress intrinsic to the experience of death and dying, the study contributes to a limited body of evidence featuring ‘untellable stories’ of suffering which cannot be relieved and which expose the reality of care for the physical and social disintegration of the dying self (Rattner, 2019). These are accounts which conflict with, and are largely excluded from, the normative discourse of ‘the good death’.

The simplistic polarisation of the ‘death awareness’ movement (Collier & Chapman, 2023; Hansford et al., 2022; Lofland, 2019) is in stark contrast to the diverse, nuanced accounts of participants which went far beyond a binary opposition between ‘good’ and ‘bad’. The discourse of the good death is a largely professional and policy construct combining ideology and pragmatism, and purveying assumptions about the purported homology between public policy and private preferences which are not borne out by the findings of this and other studies (Collier & Chapman, 2023; Hughes et al., 2008; Livne, 2019). Patients and the public risk being deceived and badly served by uncritical peddling of the idealised notion, or ‘imaginary’ of ‘the good death’, the requirement for this to be at home, and the creeping transfer of costs and responsibility for proactive preparation, forward planning, and the provision of care from professional services to the patient and their family. The professional construct of the ‘good death’ is exclusionary, unavailable, and undesirable to many dying patients and their family members. It works as a way of disciplining and constraining patient and public perspectives through the promotion of normative ‘choices’ about ‘dying well’ which fit with professional management strategies and government efforts to contain the costs of dying (Cottrell & Duggleby, 2016; Livne, 2019; Zimmerman, 2012). ‘Choice’, in such circumstances, is an illusion and the notion of ‘the good death’ an empty promise. The findings of this and other studies highlight the need to create more humane and genuinely patient- and family-centred policy based on real-world experiences rather than ideological constructs.

### Strengths and Limitations

A strength of the study was to include the perspectives of several key stakeholders from within the general population, including bereaved family members and terminally ill patients. The latter, especially, is a hard-to-reach group. However, the views of members of the general public have rarely been included in research, and the use of DDGs was a novel feature of the design. The study included a reasonable balance of men and women, but lacked wider diversity, with a concentration of older, White British and middle-class participants. The majority of patients and BFMs reported on their own experiences or that of caring for relatives with cancer. Given the recruitment of patients and some BFMs from hospices, it could be expected that these individuals may have a positive opinion of such institutions as a place of care and death, although not all reports of hospice care were favourable. All participants, especially those taking part in the DDGs, self-selected as individuals with a particular interest and willingness to talk about matters relating to death and dying, and their views may not be typical of the wider population. However, our participants were thoughtful and articulate and able to engage with the complex issues raised by the study. The move from face-to-face interviews to online data collection worked well and enabled us to recruit participants from a much wider social and geographical range than would otherwise have been able to take part including, for example, people with limited mobility or who would have been unavailable while at work. However, the COVID-19 lockdown restrictions also made recruitment of participants from some groups more difficult as we could not visit groups, events, and organisations directly to introduce the study and distribute information. This hindered our ability to reach younger people, those from different ethnic groups, and men.

## Conclusion

The normative version of the ‘good death’ finds recognition in participants’ accounts, which included descriptions of a relative dying relatively quickly at an advanced age, being accompanied by others, and free from pain. From a personal perspective, there was a strong theme of the preferred death being sudden and unexpected rather than planned and anticipated. However, the desire to avoid the trauma and distress that sudden deaths would cause to others rendered them socially undesirable and underlined the extent to which dying was experienced as relational rather than a matter of personal choice and autonomy. Participants wished to protect others from pain and distress that might be occasioned by their own dying. Dying persons and their informal caregivers made decisions based on their perception of the consequences these would have for others and in an effort to reduce the burden and distress intrinsic to the dying process. There is a dissonance between the romantic idealisation of the ‘good death’ and the reality of death and dying for many. The construct of the good death focuses on the individual rather than their social circumstances and relationships. It assumes sufficient economic, social, material, and health care resources are available to support the considerable challenges of caring for a person dying, especially at home. It also assumes that palliative and end-of-life care is available, and has the means, to enable dying persons to be in comfort and free from pain. However, our study included many accounts of distressing and protracted deaths involving great pain and suffering for all concerned. Sometimes, this was perceived to be the consequence of professional care that was inadequate and inaccessible. At others, it was attributed to the brutal and intractable nature of terminal disease which was ‘beyond the reach of palliative care’. The study findings highlight the tremendous challenges faced by families caring for dying patients at home. They raise questions about the feasibility – and desirability – of continuing current policy to promote home as the default place of death and what it is reasonable to ask family members to do in providing increasingly demanding care. The resources required to support the care of dying patients whatever the setting need to be acknowledged and current end-of-life care policy and funding radically reappraised.
